# General practitioners’ experiences of being involved in local public health work in Norway. A qualitative study

**DOI:** 10.1080/02813432.2022.2144958

**Published:** 2022-11-15

**Authors:** Dag-Helge Rønnevik, Betty Pettersen, Aslak Steinsbekk, Anders Grimsmo

**Affiliations:** a Department of Public Health and Nursing, Faculty of Medicine and Health Science, Norwegian University of Science and Technology (NTNU), Trondheim, Norway; b Municipality of Trondheim, Trondheim, Norway

**Keywords:** General practitioner (GP), health promotion, disease prevention, public health, population health, qualitative research

## Abstract

**Aim:**

The aim was to explore how general practitioners experienced being involved in local public health work and how they worked with prevention and health promotion clinically after the introduction of the Public Health Act in 2012.

**Design, setting and subjects:**

Qualitative study with focus groups interviews with 18 GPs from different municipalities in Norway.

**Results:**

The GPs said that they either had not at all or only to a limited extent been involved in local public health work in their municipalities. They reported finding it hard to prioritize individual disease prevention and health promotion in their clinical work. GPs thought of health promotion as something that mainly concerned healthy people at a group level.

**Conclusions:**

Based on the experiences of the GPs in this study, there is a gap between governmental expectations to the role of GPs in public health, and how it works in practice.

**KEY POINTS**
- With the Norwegian Public Health Act launched in 2012, GPs were expected to contribute to better population health in their clinical work and as data providers to local public health surveillance.
- The GPs interviewed in this study said they had not been involved in local public health work, and they found it hard to give disease prevention and health promotion priority in their clinical work.
- GPs expressed various perceptions of what prevention and health promotion entails.

## Introduction

Norwegian authorities have made important efforts to translate the Health in All Policies strategy (HiAP) of WHO into national policies [1,2]. The Norwegian Public Health Act (PHA) was launched in 2012 as part of a care coordination reform aiming at ‘more prevention and less treatment’ [3]. A main feature of the PHA was to place responsibility for local public health work in the political and managerial bodies, rather than in the health sector alone.

Internationally there is a growing interest in the role of primary care in public health. Collaboration between primary care and public health may result in applying a population perspective to clinical practice, identifying and addressing community health problems and strengthening prevention and health promotion (P&HP) [4]. In Europe, Norway scores medium in the achievement of primary care structure and service delivery [5], and P&HP has fallen behind treatment of diseases in Norway as well as in other European countries [6]. However, a recent report has shown that this has improved, e.g. with a 24% reduction in so-called avoidable deaths between 2009 and 2019 [7].

A key measure in the PHA for all municipalities was to monitor health in the population in a public health overview document every fourth year, with an annual update, to be presented for political review and decision-making. Public health surveillance is a critical tool for understanding a community’s health issues, and as part of implementing an evidence-based approach in public health. It provides the epidemiological foundation for modern public health practice [8]. The public health overview document is to be made by every Norwegian municipality and is expected to be based on national statistics as well as local data. This includes demographics, upbringing and living conditions, physical, biological, environmental and social conditions, injuries and accidents, health related behavior and indicators of health and wellbeing. Guidelines for making the document have been made available, together with databases with relevant indicators, courses and some guidance capacity [9].

A report from 2015 concluded that making an overview document required more resources, time, expertise and capacity than the municipalities had at their disposal [10]. Municipalities have been criticized for not utilizing local data in their public health overviews [11], and they are still in the process of legitimizing HiAP [12]. Still, the share of municipalities having made overview documents has increased to 90% in 2017, and municipalities have established cross-sectoral public health groups and engaged public health coordinators [13].

Collecting data and experiences from local health care services have been pointed out as important to create useful overview documents. The government has expressed ambitions in the PHA to include general practitioners (GPs) as data-suppliers in this process. Furthermore, both legal frameworks, national policies and professional guidelines describe and confirm the GPs’ role in individual P&HP in the joint effort of strengthening the health of the population [14,15].

GPs are in a position to balance clinical care and social interventions with the possibility of advocating their patients, addressing social determinants of health and structural causes of disease [16,17]. Their involvement in case-finding, early intervention and disease prevention, advising lifestyle-changes, empowering and supporting patients before they develop serious health problems, as well as detecting and assisting patients with low socioeconomic status, is acknowledged as important in P&HP [18]. According to the Regulation of the GP scheme, GPs must offer preventive interventions to patients at risk, and (if regulated in their contract with the municipality) make contributions to local public health work [19].

Organizational factors, funding and leadership have been found to influence collaboration between primary care and public health [20]. A WHO study, looking at integrating public health initiatives into PHC settings, found a big gap between ambitions and real life [21]. Previous Norwegian studies have shown that collaboration between GPs and local health authorities is weak, and that the GP scheme is hard to manage for the municipalities [22]. Furthermore, Norwegian GPs have placed P&HP at the bottom, when ranking what professional activities they find most meaningful [23].

The aim of this study was therefore to explore how general practitioners experienced being involved in local public health work and how they worked with prevention and health promotion clinically after the introduction of the Public Health Act in 2012.

## Materials and methods

### Study design

This was a qualitative study with semi-structured focus group interviews conducted between April and October 2017. The study was approved by The Norwegian Centre for Research Data (project # 52922). All the informants signed an informed consent. The Consolidated criteria for Reporting Qualitative research checklist (COREQ) was consulted for reporting the study [24].

### Setting

The superior responsibility for public health rests with the Royal Norwegian Ministry of Health and Care Services, while the municipalities are responsible for implementing cross-sectoral public health interventions locally and ensure that they are knowledge-based, systematic and long-term orientated.

Norway has a semi-decentralized health system, with four Regional Health Authorities (RHAs) being responsible for specialist care and 356 municipalities responsible for primary care and social service, including GPs [25]. The municipalities enjoy a great deal of freedom in organizing health services including public health. Norwegian GPs are mostly self-employed within a fee-for-service system, and operate under a contract with the municipalities they practice in. From 2001, there has been a regular GP scheme, where each inhabitant has access to a regular GP, and virtually the entire population (>99%) are registered with their regular GP.

### Participants and recruitment

The aim was to include GPs working both in rural and urban areas in Norway, with variation in age, gender and the size of the municipality they worked in. To recruit GPs, we chose to target different settings where GPs were gathered in groups and where it was likely that the intended variation in the inclusion criteria were met. These types of gatherings were identified through collegial networks. One group consisted of members from a large city in Central Norway who had met for a long time on a regular basis to discuss issues related to family medicine. The other group in Central Norway participated in a workshop arranged by local health authorities on emergency services in a regional setting of many municipalities. The last group came from two medium sized municipalities in Western Norway who took part in a workshop arranged by the authors on new ways of organizing and managing GP practices.

### Data collection

Data was collected through semi-structured focus group interviews [26], which were audiotaped and transcribed verbatim. The interviews lasted on average 90 min. The interview guide (Box 1), was developed based on research literature and documents related to the Norwegian GP scheme and the PHA. It was discussed with a group of 20 stakeholders interested in this topic

Box 1. Interview guide

Themes in the interview guide

- Experiences with public health in general
  - Experiences from public health work in general?
  - Public health work impact on your work as a GP?
  - Themes within public health of special interest?
- Organization and roles
  - Did you ever take part in public health work?
  - Are there any obstacles in the way of doing more disease prevention and health promotion in your clinic?
- Experiences with the overview document
  - Experiences with the overview document?
  - What kind of data should be in this overview?
  - What kind of data from GP clinics could be integrated?
  - How would you like to deliver data to the overview document?

### Analysis

To analyse the data, the interviews were read through to identify meanings and patterns. The authors had different approaches to how they analysed the qualitative data, and therefore we describe in detail what we did. Initially six main themes were identified (no role in in public health work, municipal public health efforts not readily available, focus individual-oriented prevention, barriers and facilitators, terminology and role of chief medical officer). DHR and BP identified meaning units which were sorted according to the initial main themes which then were revised in an iterative process. Table 1 shows an example of how the data was coded. The software programs NVivo and MindManager were used to manage the data.

**Table 1.** Example of the analytic process including meaning unit, code, sub-category and theme.

Meaning unit	Code	Sub-category	Main theme
We mostly get sick people in our office. The only healthy people I see on a regular basis, are pregnant women. In that case I guess we do some public health work.	Public health is only about healthy people	Public health work does not concern the GPs	Different perception of concepts

## Results

Three group interviews with 18 GPs were conducted (Table 2). Exact data on years in general practice were not collected, but varied from a few years to more than 30 years. The intended variation in age, gender and municipalities were reached.

**Table 2.** Characteristics of the informants (*N* = 18).

Characteristic	Number
Age (mean, range)	48 (25–70)
Gender:	
Male	11
Female	7
Municipal size	
<5,000 inhabitants	3
5,000 to 20,000 inhabitants	12
>20,000 inhabitants	3

The findings were categorized into three main themes; the participation of GPs in local public health, P&HP in clinical practice, and different perception of concepts regarding health promotion.

### GPs participation in local public health

Most of the GPs expressed limited knowledge about the Norwegian PHA of 2012 and who were responsible for coordinating public health work in their municipality.

There were various opinions about and attitudes to participation in public health work. One GP expressed lack of connection between his role as a clinician and public health work. Some were satisfied with not being involved, while others expressed concerns about being left out. Thus, some of the informants seemed to be conflicted about it, like this GP:

I do not choose to be passive, but I have more than enough to cope with in my daily work. I experience public health and health promotion as something that is not natural for me to take part in.

None of the informants had experienced being directly involved in the municipality’s public health work. They neither referred to participation in the process of making the public health overview document, nor to delivering quantitative or qualitative data from their practices. Some of them were positive to deliver data from their electronic health record systems (EHR) for public health purposes, provided time and payment. Others were skeptical, characterizing the overview document as ‘just some kind of statistics’ with no value for daily work, and that integrating qualitative information from GPs in the overview document could be viewed as ‘guesswork’ with no value. Nevertheless, some felt they could contribute more, if consulted:

I don’t think the work with the overview document is being done in a good way. They could have obtained more information from us. When GPs have been around for a while, they have first-hand knowledge about the health of many generations.

On the other hand, some of the GPs expressed relief that they could lean on the local Chief medical officer (CMO) to perform this work.

When asked, none of the GPs said they had access to routines for how GPs could report observed failure in health care for vulnerable patients, suggest improvements in health care services or start-up of new interventions at population level.

One GP described how the introduction of the GP regular scheme in 2001, with the fee-for-service system, brought about a shift in his relation to public health:

After 2001, I haven’t been involved in public health. Earlier on, when I had regular salary and was employed by the municipality, I took part in different workgroups and the like. At that time, I had a kind of meta-perspective on health and health care. But now, after the GP-scheme, we are only doctors in the clinic.

### Prevention and health promotion in clinical practice

The participants acknowledged disease prevention as an integral part of their daily work, exemplified by prescribing contraception, vaccinations and advising lifestyle changes. One GP compared GPs’ work to fishermen’s work; with their nets they catch high-risk patients, the rest they let go.

To enhance P&HP, some GPs suggested employing nurses in the clinics, to increase quality of care for patients with chronic conditions while others desired more teamwork with other professions, and opportunities to run group sessions. Several GPs expressed that a better overview of their population would secure better care. Except for one, none had technical solutions to extract the necessary statistics from their EHR systems. Many held up continuity in care and broad knowledge of their patients to be of great value, enabling early prevention and support. Like this GP:

If you see an obese patient, and you know that there were diabetes and heart disease in previous generations, then there is an increased risk. You sit on a lot of local knowledge, and you know your population quite well.

Some GPs expressed that P&HP was not worth prioritizing, describing it as ineffective and utopian, and not part of GPs’ main responsibility, described to be treating diseases. Some pointed at risk assessment as leading to medical overtreatment of healthy people. They also described barriers to prioritizing P&HP as lack of guidelines, inadequate interdisciplinary competency and capacity in their clinics, and distanced or unknown services to refer to and to cooperate with. The informants also emphasized that available information about low threshold services in their community is crucial to fulfil their role in coordinating health care for their patients. Still, the most commonly mentioned barriers were heavy workload and lack of time, both in general and within consultations:

It takes time to motivate patients to make lifestyle changes – time that I feel I don’t have, because I don’t want to give priority to it.

Deficient financial incentives were also an often-mentioned barrier, with reference to a fee-for-service scheme based mainly on treating diseases, rather than P&HP interventions. The GPs told that they did not receive full compensation for attending meetings outside working hours, and that they could not reduce their lists without losing money. Regarding the use of tariffs, one of the doctors stated: ‘I think it [the fee for service system] controls our behavior to a large extent’.

Regarding lifestyle changes, GPs referred to patients’ different levels of knowledge, attitudes and motivation as challenging. They also called for strengthened knowledge and better skills about P&HP among GPs:

There is no consensus regarding how to perform disease prevention. We need more information about what kind of advice we should give to different groups of patients.

### Differences in perception of concepts

The term *health promotion* was hardly mentioned by the GPs when discussing the clinical context, while the terms *primary and secondary disease prevention* was frequently used. Some GPs described GPs to have a dualistic image of the population as either sick or healthy people. A young doctor expressed the following:

We mostly get sick people in our office. The only healthy people I see on a regular basis, are pregnant women. In that case I guess we do some public health work.

Most of the participants described conditions like high blood-pressure, pre-diabetes and osteoporosis as diseases rather than as risk-factors, and accompanying interventions as treatment, not as prevention. When challenged on these views during the interviews, discussions on the link between individual medicine in the clinic and public health arose. In one of the groups, some of the GPs said they had gained a new understanding when GPs individual P&HP interventions were acknowledged as a legitimate part of the joint effort to improve the health of the population:

I guess you could say that if we get one patient healthier, or prevent her/him from getting worse, it is a small piece in the big picture that eventually will lead to a healthier population.

## Discussion

The GPs in this study had no experience of being involved by the municipalities in public health work or the process of making public health overview documents. They found it hard to prioritize disease prevention and health promotion in their clinical work, due to time constraint and financial incentives like fee-for-service which promotes treatment. The GPs own understanding was that they did not work with public health as they understood it. Rather they saw themselves as working with treatment of individuals and that this was a contribution to improving the health of the population.

### Strengths and weaknesses

The strengths of this study include variation in GP characteristics which was a likely cause of the breadth of the discussion and many different points of views. The authors’ background as GPs and CMOs provided important insight when designing the study.

There were some noteworthy limitations. The sample was from two regions in Norway, but as there were good variation from rural and urban municipalities this is not considered to hamper generalizability. It was consciously chosen not to include informants with responsibility for public health work in the municipalities like chief medical officers, and the findings can thus not be transferred to this group. The GPs seemed to have differing understandings of central concepts like health promotion and disease prevention. This may have led the GPs to misunderstand each other during the interviews by e.g. agreeing to something another GP said due to interpreting the speaker to use the same understanding as her-/himself. Furthermore, the authors’ own understanding of these concepts could influence the interpretation of what was said. To reduce this limitation, care was taken not to take the terms used at face value and to have in-depth discussions between the authors about their own understanding.

### A distant relationship between GPs and public health

Our results indicate that GPs have limited insight and involvement in local public health work in their municipalities in general, and in the process of making the legally required overview documents. This may be a sign of the traditional gap between public health and primary health care still being there, as described by others [27].

Some of the GPs had a negative attitude towards being involved in public health. This is supported by a scoping study from 2017, concluding that many GPs do not take a population approach in their clinics [28]. Lack of time was reported as the main reason for this by the GPs in our material. This can also be seen in light of a shift in the introduction of the GP scheme in 2001 where several doctors chose to switch from being a public employee to working in private clinics [29,30]. The regular GP scheme in Norway has lately been under pressure, with recruitment and retaining challenges and increased workload [31]. GPs have expressed concerns for the future, particularly with regards to GPs’ health and motivation [32]. If health promotion and prevention take longer time and has weaker financial incentives in the fee-for-service system, the current situation can make it more difficult for GPs to prioritize this type of work.

Still, some GPs in this study were positive to provide quantitative data directly to local public health surveillance purposes. This is in accordance with ambitions from the government, the PHA and the GP regulation, and can help realize the synergies described by Shahzad et.al. [4]. Even with increasing ability to extract data from EHR systems, there are issues related to GPs’ willingness to expand todays clinical model to collect and process new types of data and to serve public health goals [33]. Internationally, quantitative data from primary care sentinel networks have been used for research and epidemiological surveillance. In Norway, computer-based data from GPs has been demonstrated useful for planning and evaluation, also for public health purposes [34].

Municipalities have been encouraged to collect qualitative data from personnel in primary care [35]. But none of the GPs interviewed had been consulted by the municipality to share their experiences or ideas on the health of the population or the organization of health care services. This finding is contrary to the belief of synergies from interwinding public health and general practice. It has been suggested that qualitative data is needed for population health management and policy making, complementing and turning quantitative data into policy-relevant stories [36]. However, the GPs we interviewed seemed to have little trust in such use of data, as they thought their personal opinions would not be included.

### Disease prevention and health promotion under pressure in general practice

In the biomedical model, there has traditionally been a distinction between disease prevention and health promotion based on dichotomization between sick and healthy people. It is possible, however, to use health-promoting methods and theories in a general medical setting at the individual level [37]. Our informants did not relate to the term ‘health promotion’, but focused on primary and secondary disease prevention and saw this as an integrated part of their daily work. Diabetes, obesity, smoking, hypertension, and hypercholesterolemia were mentioned as some of the most important conditions. This is in accordance with other studies [38], and indicates that the biomedical model still dominates GPs perception of P&HP.

The GPs highlighted their position as coordinators for their patients, and some also searched for opportunities to increase team activities with other health professions in their clinics, in line with present health policies. They also called for changes at a system level to facilitate P&HP, such as more time, stronger financial incentives, better access to low threshold referral services, better guidelines and the opportunity to increase their own skills and competence. Other researchers have reported how competing demands make it hard for GPs to make P&HP a priority [39,40]. References to time pressure noted in other studies was a recurrent theme also in our interviews.

Some GPs were negative towards P&HP, describing it as ineffective and utopian. In 2014 Rubio-Valera et al. identified factors influencing the implementation of P&HP activities in primary care, and fitted them into a five-level ecological model: intrapersonal factors, interpersonal processes, institutional factors, community factors and public policy [41]. One of the main factors to succeed implementing P&HP, they concluded, is related to the beliefs, attitudes and motivations of professionals at the intrapersonal level. They also pointed at the importance of skills required to carry out P&HP activities, and support from managers and patients to implement P&HP. Our findings suggest variation within P&HP provided by Norwegian GPs, which from a governmental point of view is unwanted.

### Terminological barriers

Our informants hardly mentioned the term health promotion. Some of them divided the population into either sick or healthy persons, leading to the conclusion that health promotion only concerns healthy persons. This was surprising, since the term is well known and in use in both Norwegian and international GP-societies. This understanding is not in line with the core values of the Nordic Association of General Practitioners presented in 2021, saying that disease prevention and health promotion are integrated into GPs’ daily activities [42].

According to a resent Norwegian study, the use of common terminology is a central facilitating factor when implementing the PHA at the local level [43]. The different perception of concepts in medicine on the one hand and public health on the other, is therefore likely to obstruct GPs’ involvement in public health. It might also impede them from using theories and methods of health promotion in their practice [44]. On the other hand, our findings may indicate that GPs perform more P&HP than both they themselves and others acknowledge, though they use other words to describe it.

We experienced a shift in the conversations when introducing the term ‘population health’. This made the GPs make a connection between their efforts with individual patients and public health work in general. Population health is seen as a hybrid of public health and clinical medicine, drawing from the traditions of both approaches [16]. Hence, using this term may facilitate the synergies between primary care and public health.

## Implications for practice, policy and research

**Figure 1.**
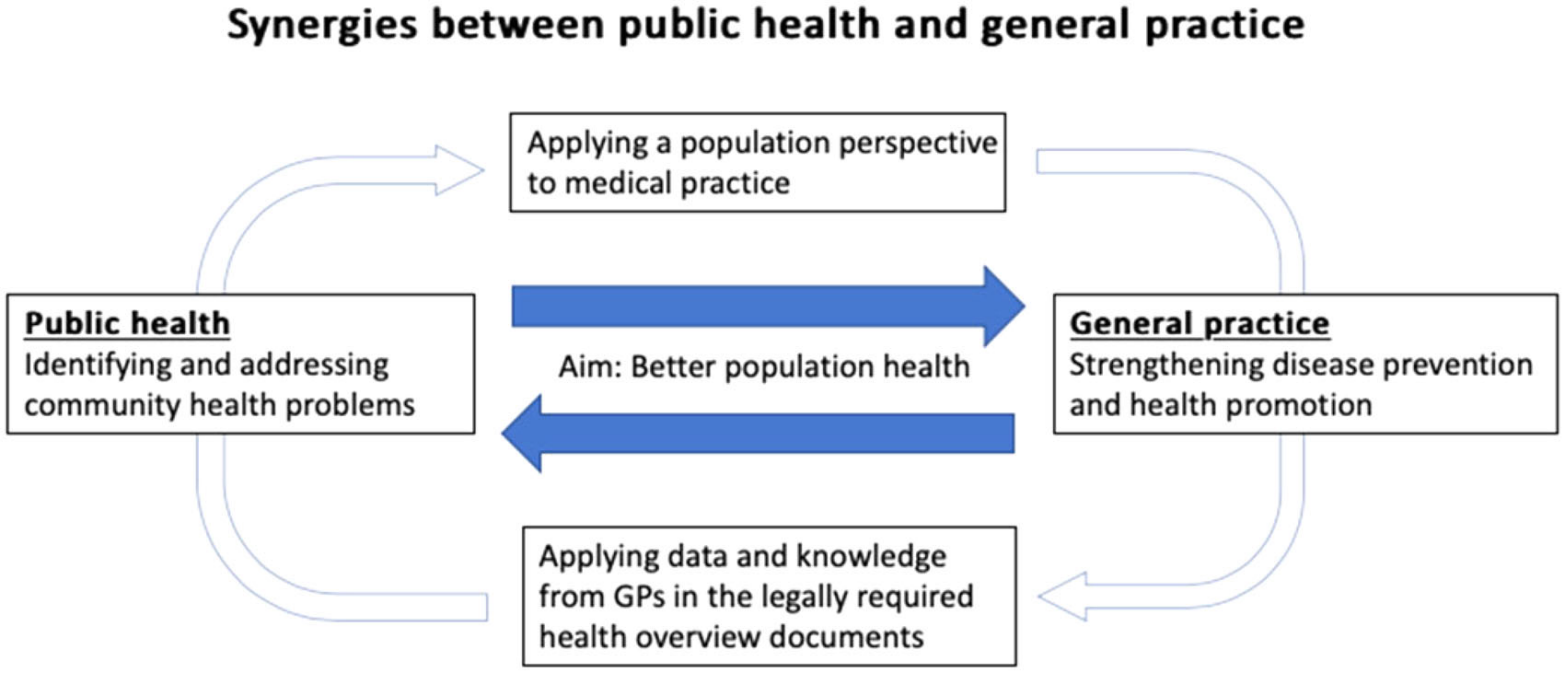
GPs are in a dual position to apply a population perspective to their medical practice, at the same time as they could contribute to local public health work as data-providers (both quantitative and qualitative data). This represents a possible connection between clinical medicine and public health at the local level.

A main finding in this study was that the GPs did not have a clear understanding of their expected role in local public health work and that the municipalities did not involve them. Thus, to illustrate for GPs, officials in municipalities and others the ambitions in the PHA to strengthen the synergies between clinical medicine and public health, we have made a figure that shows the two complementary roles of GPs (Figure 1). Firstly, GPs can apply a population perspective in their practice, which includes acquiring knowledge of and the use of the public health overview document from their municipality. Secondly, GPs can supply their municipalities with quantitative and qualitative data to be used in the legally required health overview documents. The common aim is to improve the health of the population.

For this interaction to work, our findings indicate that a stronger emphasis on the use of methods and theories within health promotion at the individual level, both in the medical education and in the GP specialization program, is needed. Furthermore, the municipalities should acknowledge the GPs efforts and take a role in activating the GPs in local public health work. The respondents indicated that the CMOs could have a role as a local facilitator and liaison between GPs and municipalities. If the synergies between clinical medicine and public health are to be realized, this should build on shared ideology, theoretical orientation and terminology. Also institutional barriers such as workload, financial incentives (including the set-up of the fee-for-service system) and capacity needs to be addressed.

## Conclusion

Given the experience of the GPs in this study, there is a gap between the national ambitions to include GPs in local public health work, and how this is done in practice.
